# Histiocytic pleural effusion: the strong clue to malignancy

**DOI:** 10.1186/s12957-021-02296-1

**Published:** 2021-06-16

**Authors:** Ganghee Chae, Jae-Bum Jun, Hwa Sik Jung, Chui Yong Park, Jin Hyoung Kim, Byung Ju Kang, Hyeon Hui Kang, Seung Won Ra, Kwang Won Seo, Yangjin Jegal, Jong Joon Ahn, Sang Hyuk Park, Taehoon Lee

**Affiliations:** 1 Department of Pulmonary and Critical Care Medicine, Asan Medical Center, University of Ulsan College of Medicine, Seoul, Republic of Korea; 2 Division of Infectious Diseases, Department of Internal Medicine, Ulsan University Hospital, University of Ulsan College of Medicine, Ulsan, Republic of Korea; 3 Division of Pulmonary and Critical Care Medicine, Department of Internal Medicine, Samsung Medical Center, Sungkyunkwan University School of Medicine, Seoul, Republic of Korea; 4 Division of Pulmonary and Critical Care Medicine, Department of Internal Medicine, Ulsan University Hospital, University of Ulsan College of Medicine, 877 Bangeojinsunhwando-ro, Dong-gu, Ulsan, 44033 Korea; 5 Department of Laboratory Medicine, Ulsan University Hospital, University of Ulsan College of Medicine, Ulsan, Republic of Korea

**Keywords:** Histiocytes, Thoracentesis, Pleural effusion, Exudate, Malignancy

## Abstract

**Background:**

There have been many studies on the clinical characteristics of neutrophilic, lymphocytic, and/or eosinophilic pleural effusion. While caring for patients with pleural effusion, we found that histiocytic pleural effusion (HisPE) was not uncommon. However, few studies have explored HisPE. The purpose of the present study was to determine the clinical characteristics and etiologies of HisPE.

**Methods:**

In this retrospective study, HisPE was defined as pleural fluid white blood cells comprised of ≥ 50% histiocytes. Using a clinical data warehouse, patients with HisPE among all patients aged >18 years who underwent thoracentesis and pleural fluid analysis between January 2010 and December 2019 at Ulsan University Hospital were enrolled. A total of 295 (9.0%) of 3279 patients who underwent thoracentesis were identified as HisPE patients. Among them, 201 with exudative HisPE were included. Clinical characteristics and etiologies were extracted from medical records and analyzed.

**Results:**

Among the 201 patients with exudative HisPE, the major causes were malignant pleural effusion (*n* = 102 [50.7%]), parapneumonic effusion (*n* = 9 [4.5%]), and tuberculous pleurisy (*n* = 9 [4.5%]). In the 102 patients with malignant pleural effusion, the main types of cancer were lung (*n* = 42 [41.2%]), breast (*n* = 16 [15.7%]), and stomach cancer (*n* = 11 [10.8%]). Among lung cancers, adenocarcinoma (*n* = 34 [81.0%]) was the most common histology.

**Conclusions:**

The leading cause of exudative HisPE was malignancy, particularly lung cancer. Physicians should consider the possibility of malignant disease if histiocytes are predominantly present in pleural effusion.

## Background

The pleural space typically contains only a few milliliters of pleural fluid, which is not visible on imaging studies. Thus, the detection of fluid in the pleural space on imaging studies is abnormal. Many conditions are associated with pleural fluid accumulation, and the most useful method for the differential diagnosis of pleural effusion (PE) is a diagnostic thoracentesis [1, 2]. Via diagnostic thoracentesis, PE can be categorized as transudate or exudate according to Light’s criteria [3]. Transudative PE is a secondary manifestation of extrapulmonary systemic diseases that induce volume overload; therefore, further pleural fluid examination is generally not needed. In contrast, exudative PE is predominantly caused by a primary disease of the lung or pleura and requires further diagnostic investigations. Among them, the differential count of pleural fluid white blood cells (WBCs) aids differentiation of the causal diseases. Pleural fluid WBCs include neutrophils, lymphocytes, eosinophils, and other mononuclear cells such as histiocytes [2, 4, 5].

The characteristics and causes of neutrophilic, lymphocytic, and eosinophilic PE are relatively well-established [4–6]. However, although histiocytic pleural effusion (HisPE) is not uncommon in clinical practice, few studies have explored HisPE. Therefore, the aim of the present study was to determine the clinical characteristics and etiologies of HisPE.

## Methods

### Study design and population

This was a retrospective cross-sectional study. All patients aged >18 years who underwent thoracentesis and pleural fluid analysis between January 2010 and December 2019 at Ulsan University Hospital were initially collected (*n* = 3279). We defined HisPE as when histiocytes comprised ≥50% of the differential count of pleural fluid WBCs; using this definition, 295 (9.0%) of 3279 patients were identified as HisPE patients. Among the 295 patients with HisPE, Light’s criteria were used to exclude patients with transudative HisPE (*n* = 90). Those with missing data (*n* = 4) were also excluded. Accordingly, 201 exudative HisPE patients were enrolled and analyzed.

### Data collection

Initial patient data were collected from a clinical database platform in conjunction with the electronic medical records at Ulsan University Hospital (Ulsan University Hospital Information of Clinical Ecosystem [uICE]). The collected data included age, sex, smoking status, pleural fluid differential cell count of WBCs, red blood cell (RBC) count, adenosine dehydrogenase (ADA), protein and lactate dehydrogenase (LDH), and serum protein and LDH.

In order to determine the final diagnosis (i.e., causes of HisPE), a detailed chart review of individual patients was conducted. The following criteria were used for the causes of HisPE: Malignant PE was defined as when pleural fluid cytology or pleural biopsy confirmed malignancy, or when a patient was previously diagnosed with cancer and also had PE but PE could not be explained by other causes [7]. Tuberculous pleurisy was defined as when the *Mycobacterium tuberculosis* was isolated from the respiratory specimens or pleural fluid using any mycobacterial culture or molecular method, or when PE was improved by antituberculosis treatment after a clinical diagnosis of tuberculosis by a physician [8]. Parapneumonic effusion was defined as when a patient diagnosed with pneumonia had simultaneous PE and did not meet the definition of malignant PE or tuberculous pleurisy [7]. PE due to heart failure, liver cirrhosis, or renal failure was defined as when individual diseases were identified, and PE improved following treatments for the individual diseases. Miscellaneous PE referred to PE due to other uncommon specific causes (e.g., chylothorax, traumatic hydrothorax, hemothorax, acute pancreatitis, drug-induced PE, atelectasis, other infectious diseases). Lastly, idiopathic PE was defined as when the above definitions were not met.

The present study was approved by the Institutional Review Board of Ulsan University Hospital (IRB number: UUH 2020-04-028).

### Pleural fluid processing

**Fig. 1:**
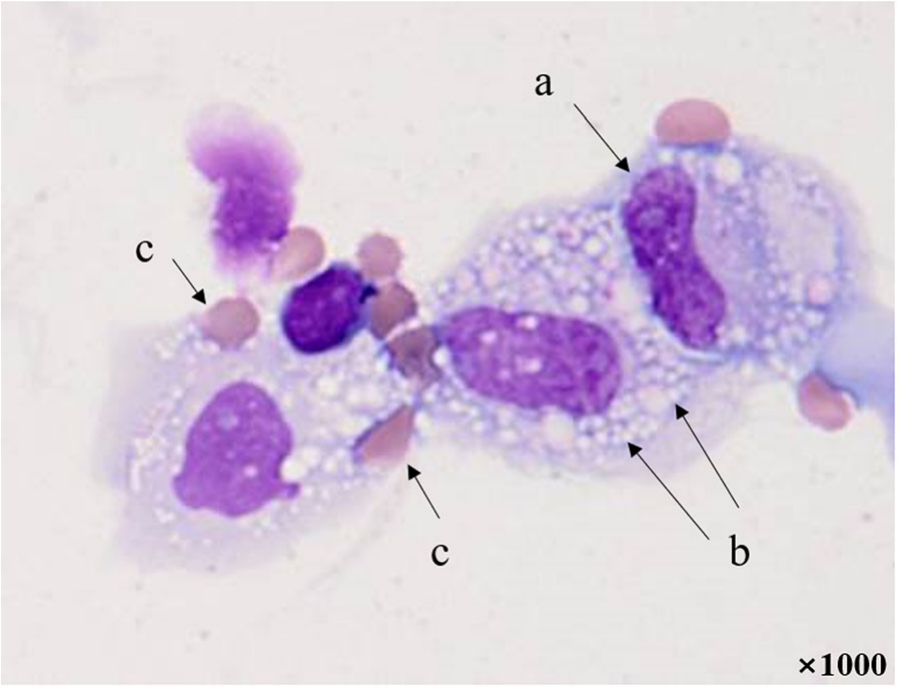
Microscopic image of histiocytes in pleural fluid (Wright-Giemsa stain, ×1000). Histiocytes can be observed as single or grouped cells, and their sizes vary considerably, ranging from approximately 15 to 100 μm in diameter. The nucleus (a) is located on one side of the cytoplasm in a distorted form or a kidney shape. The cytoplasm contains vacuoles (b) and ingested red blood cells (c) and has an unclear boundary

**Fig. 2:**
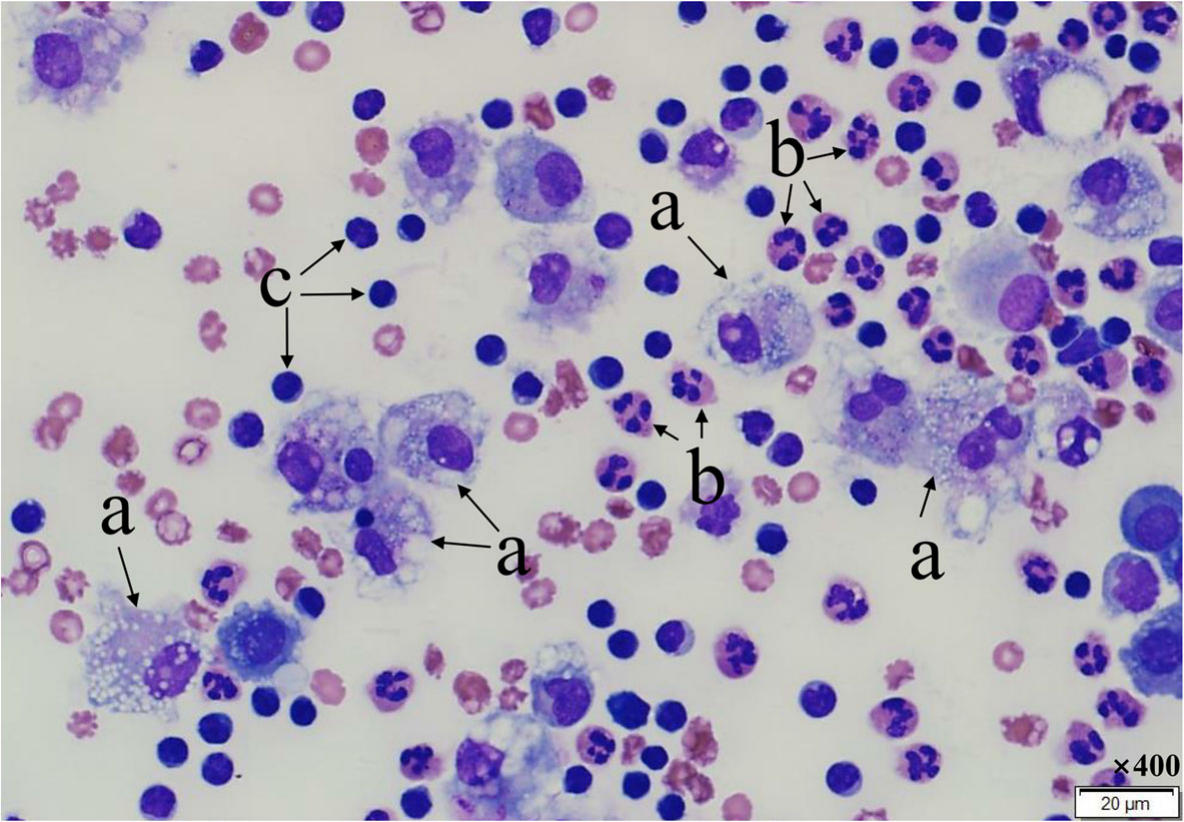
Microscopic image of white blood cells in pleural fluid (Wright-Giemsa stain, ×400). Histiocytes (a) appear as described in Fig. 1. Neutrophils (b) are 12–14 μm in diameter and appear larger than the surrounding RBCs. They have a single nucleus, which contains 2–5 lobes, and their cytoplasm has many granules. Lymphocytes (c) are small (approximately 6–9 μm) and have a spherical nucleus. The cytoplasm is small and basophilic

Pleural fluid samples were collected in EDTA tubes and immediately sent to the laboratory at ambient temperature. Manual cell counts were performed using a hemocytometer at high magnification (×400) under a light microscope, and the cell suspension of pleural fluid was adjusted to an optimal concentration (approximately 5.0 × 10^5^ cells/mL). The suspension was then cytocentrifuged at 600 rpm for 5 min (Cellspin; Hanil Science Industrial, Korea), and the preparations were stained with Wright-Giemsa stain. The differential count of WBCs was determined by counting 100 cells under a light microscope (×400). Lymphocytes, neutrophils, histiocytes, eosinophils, mesothelial cells, malignant cells, and atypical cells were differentiated (Figs. 1 and 2).

### Data analysis

Results were derived through descriptive analyses, and data are expressed as numbers (percentages) and medians (interquartile range [IQR]).

## Results

Among the 201 exudative HisPE patients, the most common diagnosis was malignant PE (*n* = 102 [50.7%]), followed by idiopathic PE (*n* = 36 [17.9%]); PE due to heart failure, liver cirrhosis, or renal failure (*n* = 24 [11.9%]); miscellaneous PE (*n* = 21 [10.4%]); parapneumonic effusion (*n* = 9 [4.5%]); and tuberculous pleurisy (*n* = 9 [4.5%]).

The baseline characteristics of exudative HisPE patients, with the exception of miscellaneous and idiopathic conditions are presented in Table 1. Patients with parapneumonic effusion were older than those with other causes. The proportions of females and non-smokers were higher among patients with malignant PE, which is thought to be due to the high incidence of breast cancer in our study. In comparison, the proportion of males was higher among those with PE due to heart failure, liver cirrhosis, or renal failure.

**Table 1:** Baseline characteristics of patients with exudative HisPE

	Malignant PE	Parapneumonic effusion	Tuberculous pleurisy	Heart failure, liver cirrhosis, or renal failure
Number	102 (50.7)	9 (4.5)	9 (4.5)	24 (11.9)
Age (years)	62.0 (52.0–74.0)	71.0 (64.0–73.0)	63.0 (54.0–79.0)	64.5 (54.0–79.0)
Sex				
Male	49 (48.0)	5 (55.6)	5 (55.6)	17 (70.8)
Female	53 (52.0)	4 (44.4)	4 (44.4)	7 (29.2)
Smoking status				
Non-smoker	64 (62.7)	4 (44.4)	3 (33.3)	9 (37.5)
Current/ex-smoker	8 (7.8)/30 (29.4)	2 (22.2)/3 (33.3)	2 (22.2)/4 (44.4)	7 (29.2)/8 (33.3)
Pleural fluid				
RBC (/uL)	3750 (740–27000)	6240 (70–11,200)	560 (250–3800)	495 (180–7110)
WBC (/uL)	460 (200–1000)	2500 (430–3680)	570 (310–720)	320 (105–560)
Histiocyte (%)	62.5 (55.0–70.0)	66.0 (53.0–78.0)	69.0 (62.0–74.0)	63.0 (55.0–77.5)
Neutrophil (%)	5.0 (2.0–12.0)	10.0 (3.0–12.0)	5.0 (2.0–6.0)	2.0 (1.0–6.5)
Lymphocyte (%)	16.5 (10.0–26.0)	7.0 (1.0–11.0)	19.0 (11.0–28.0)	16.0 (10.0–29.5)
Mesothelial cell (%)	0.0 (0.0–2.0)	0.0 (0.0–15.0)	0.0 (0.0–1.0)	0.5 (0.0–11.0)
ADA (IU/L)	24.7 (15.9–34.3)	27.0 (17.7–38.0)	71.4 (47.1–107.1)	14.5 (9.4–19.3)
Protein (g/dL)	4.2 (3.4–4.6)	4.3 (3.9–5.0)	3.5 (2.9–3.7)	2.8 (1.7–3.3)
LDH (IU/L)	305.0 (202.0–706.5)	254.0 (184.0–361.0)	207.0 (179.0–266.0)	173.0 (138.0–219.0)
Serum				
Protein (g/dL)	6.2 (5.6–6.6)	6.4 (5.6–7.3)	6.1 (6.0–6.6)	6.3 (5.5–6.7)
LDH (IU/L)	317.0 (210.0–435.0)	149.0 (128.0–212.0)	331.0 (191.0–488.0)	492.0 (277.0–685.0)

Data are presented as n (%) or median (interquartile range)

*HisPE* histiocytic pleural effusion, *PE* pleural effusion, *RBC* red blood cell, *WBC* white blood cell, *ADA* adenosine deaminase, *LDH* lactate dehydrogenase

In the pleural fluid analysis, there was no apparent difference in the RBC count, but the WBC count was higher among patients with parapneumonic effusion than those with other causes. Additionally, in the differential count of pleural fluid WBCs, the parapneumonic effusion group had a slightly higher proportion of neutrophils than lymphocytes (histiocytes 66.0%, neutrophils 10.0%, lymphocytes 7.0%). Meanwhile, patients with malignant PE and tuberculous pleurisy had a higher proportion of lymphocytes than neutrophils (malignant PE: histiocytes 62.5%, neutrophils 5.0%, lymphocytes 16.5%; tuberculous pleurisy: histiocytes 69.0%, neutrophils 5.0%, lymphocytes 19.0%). The proportion of mesothelial cells was very low in all groups. In addition, the pleural fluid ADA level was markedly higher among those with tuberculous pleurisy than those with other causes (IU/L, median [IQR]: tuberculous pleurisy, 71.4 [47.1–107.1]; malignant PE, 24.7 [15.9–34.3]; parapneumonic effusion, 27.0 [17.7–38.0]; heart failure, liver cirrhosis, or renal failure, 14.5 [9.4–19.3]). Further, the pleural fluid protein level was markedly lower in those with PE due to heart failure, liver cirrhosis, or renal failure relative to those with other causes (g/dl, median [IQR]: heart failure, liver cirrhosis, or renal failure, 2.8 [1.7–3.3]; malignant PE, 4.2 [3.4–4.6]; parapneumonic effusion, 4.3 [3.9–5.0]; tuberculous pleurisy, 3.5 [2.9–3.7]). The pleural fluid LDH level was higher in those with malignant PE than in those with other causes (IU/L, median [IQR]: malignant PE, 305.0 [202.0–706.5]; parapneumonic effusion, 254.0 [184.0–361.0]; tuberculous pleurisy, 207.0 [179.0–266.0]; heart failure, liver cirrhosis, or renal failure, 173.0 [138.0–219.0]). There was no apparent difference in the serum protein level, but the serum LDH level was higher among those with PE due to heart failure, liver cirrhosis, or renal failure than those with other causes (IU/L, median [IQR]: heart failure, liver cirrhosis, or renal failure, 492.0 [277.0–685.0]; malignant PE, 317.0 [210.0–435.0]; parapneumonic effusion, 149.0 [128.0–212.0]; tuberculous pleurisy, 331.0 [191.0–488.0]).

**Fig. 3:**
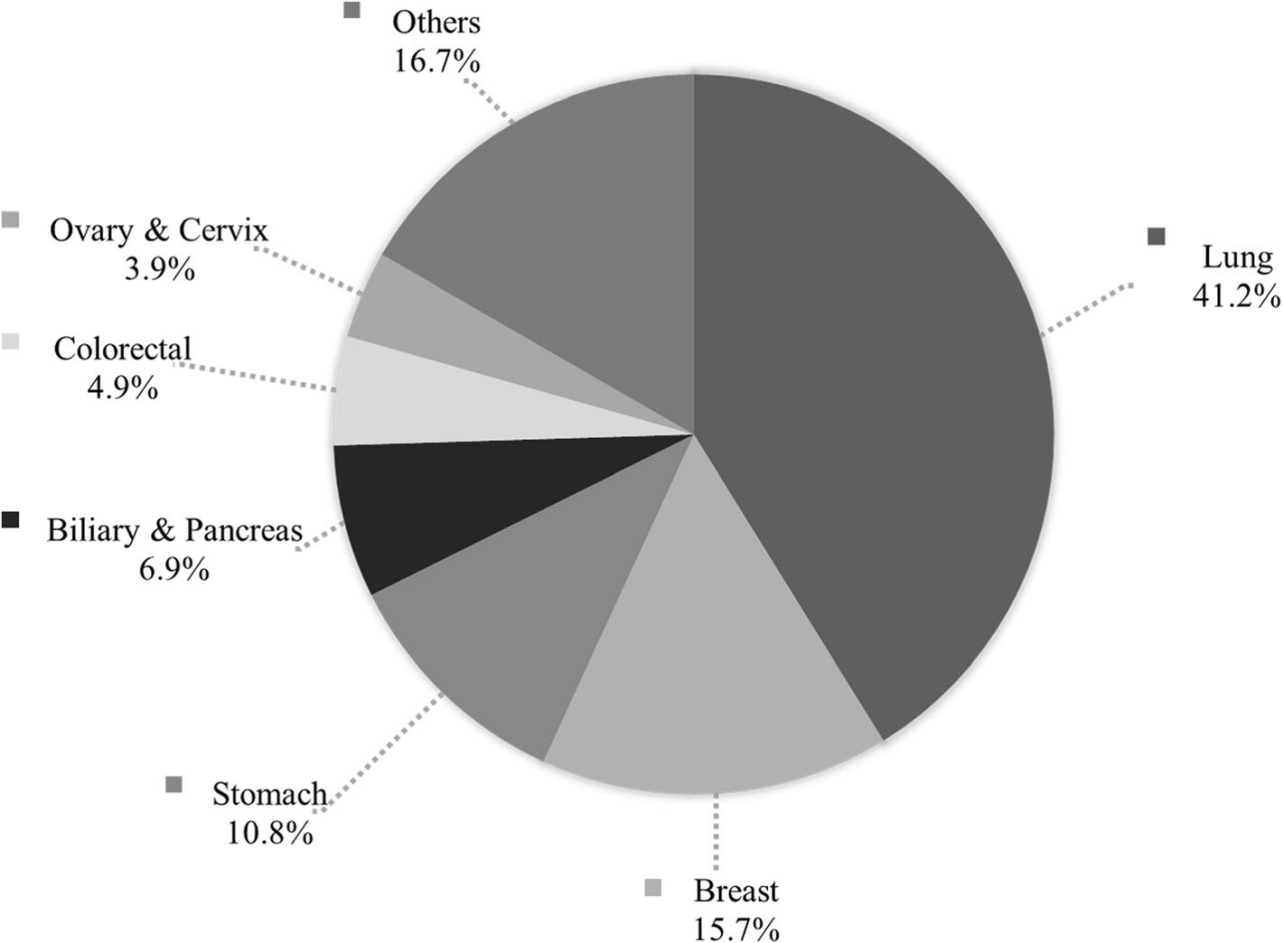
Distribution of the causes of malignancy in histiocytic pleural effusion (*n* = 102). Among the 102 patients with malignancy and histiocytic pleural effusion, the causes of cancer were as follows: lung cancer (*n* = 42 [41.2%]), breast cancer (*n* = 16 [15.7%]), stomach cancer (*n* = 11 [10.8%]), biliary and pancreas cancer (*n* = 7 [6.9%]), colorectal cancer (*n* = 5 [4.9%]), ovary and cervix cancer (*n* = 4 [3.9%]), and other malignancies (*n* = 17 [16.7%])

Regarding cancer types among the 102 patients with malignant PE, lung cancer (*n* = 42 [41.2%]), breast cancer (*n* = 16 [15.7%]), and stomach cancer (*n* = 11 [10.8%]) were the most common causes (Fig. 3). Among lung cancers, which was the most common cause of malignant PE, adenocarcinoma (*n* = 34 [81.0%]) was the most common histology, followed by small cell lung cancer (*n* = 4 [9.5%]), squamous cell carcinoma (*n* = 3 [7.1%]), and non-small cell lung cancer—not otherwise specified (*n* = 1 [2.4%]).

Thoracentesis for exudative HisPE patients was typically performed in the general ward (66.7%) and emergency room (30.6%). There was no apparent difference in the location according to the causal disease (Table 2). In addition, when confirming the component to which Light’s criteria were applied, both the protein and LDH criteria were mostly satisfied among those with malignant PE (60.8%), parapneumonic effusion (66.7%), and tuberculous pleurisy (66.7%). In comparison, those with PE due to heart failure, liver cirrhosis, or renal failure generally satisfied only the LDH criteria (66.7%) (Table 3).

**Table 2:** Location of thoracentesis in patients with exudative HisPE

	Malignant PE (*n* = 102)	Parapneumonic effusion (*n* = 9)	Tuberculous pleurisy (*n* = 9)	Heart failure, liver cirrhosis, or renal failure (*n* = 24)	Total (*n* = 144)
General ward	74 (72.5)	6 (66.7)	4 (44.4)	12 (50.0)	96 (66.7)
Intensive care unit	0 (0)	1 (11.1)	0 (0)	3 (12.5)	4 (2.8)
Emergency room	28 (27.5)	2 (22.2)	5 (55.6)	9 (37.5)	44 (30.6)

Data are presented as n (%)

*HisPE* histiocytic pleural effusion, *PE* pleural effusion

**Table 3:** Component to which Light’s criteria are applied in exudative HisPE

	Malignant PE (*n* = 102)	Parapneumonic effusion (*n* = 9)	Tuberculous pleurisy (*n* = 9)	Heart failure, liver cirrhosis, or renal failure (*n* = 24)	Total (*n* = 144)
Only protein criteria^a^	19 (18.6)	1 (11.1)	1 (11.1)	5 (20.8)	26 (18.1)
Only LDH criteria^b^	21 (20.6)	2 (22.2)	2 (22.2)	16 (66.7)	41 (28.5)
Both protein and LDH criteria	62 (60.8)	6 (66.7)	6 (66.7)	3 (12.5)	77 (53.5)

Data are presented as n (%)

*HisPE* histiocytic pleural effusion, *PE* pleural effusion, *LDH* lactate dehydrogenase

^a^The ratio of pleural fluid to serum protein is over 0.5

^b^The ratio of pleural fluid to serum LDH is over 0.6 or the absolute pleural fluid LDH level is over 2/3 of the upper normal limit

## Discussion

This study aimed to determine the clinical characteristics and etiologies of HisPE. We found that HisPE was not uncommon (9.0%) among patients with PE, and >50% of exudative HisPE cases were caused by malignancy (50.7%). The major malignancies were lung cancer, breast cancer, and stomach cancer. Thus, physicians must consider the possibility of underlying cancer if they encounter HisPE.

In a previous study on the cellular content of pleural fluid from patients with normal pleura who were undergoing thoracoscopy for hyperhidrosis, approximately 75% of the cells in the pleural fluid were macrophages, belonging to histiocytes [9]. That is, histiocytes comprised a considerable proportion of normal pleural fluid. However, the characteristics and etiologies of patients with HisPE due to pathologic conditions have not been studied. In the present study, we defined HisPE as when histiocytes comprised 50% or more of the pleural fluid WBCs. In our clinical experience, we have found that HisPE is not uncommon, and felt that it is associated with malignant PE. We presumed that pleural metastasis of cancer would trigger immune responses and induce reactive histiocytic proliferation [10]. Thus, we started this study because of this insightful experience.

Previous studies investigating HisPE consist of only case reports. One such report observed proliferating histiocytes in the pleural fluid with no palpable lymphadenopathy or organomegaly; this was an atypical case of Rosai-Dorfman disease (also known as sinus histiocytosis with massive lymphadenopathy), which involves the overproduction of non-Langerhans sinus histiocytes [11]. Another case report described histiocytic proliferation mimicking metastatic signet ring cell adenocarcinoma and highlighted the importance of differentiation between histiocytic proliferation and signet ring cell carcinoma [12]. There was also a report of histiocytic sarcoma presenting with HisPE. In this case report, a patient presented with anterior mediastinal tumor accompanied by HisPE, and the cause of the mass was histiocytic sarcoma [13].

The term “histiocytes” was originally used to describe the large cells commonly found in the lymph nodes and spleen that were morphologically nonspecific. Currently, histiocytes are considered to be tissue macrophages that are differentiated from the monocyte lineage, including alveolar macrophages in the lung, Kupffer cells in the liver, Langerhans cells in the skin, and dendritic cells in the germinal centers of lymph nodes [14]. These cells (histiocytes) play an important role in antigen presentation, phagocytosis, and removal of pathogens and cellular debris, and can be found anywhere on the human body including the PE [15, 16].

There are two situations in which histiocytes could increase: reactive and neoplastic histiocytic proliferation [10]. Neoplastic proliferation refers to the clonal proliferation of histiocytes, such as acute/chronic myelomonocytic leukemia, acute monocytic leukemia, and histiocytic sarcoma [10]. Reactive proliferation is caused by inflammatory responses secondary to infection, autoimmune diseases, or malignancies. Granuloma is a representative reactive proliferation, wherein histiocytes fuse to form giant cells [10]. Severe inflammation may cause hemophagocytic lymphohistiocytosis via hypercytokinemia [16].

The causative mechanism of reactive histiocytosis is poorly understood. It is presumed that histiocytes, which are antigen-presenting cells, are likely increased by antigenic or microbial stimuli [14]. In the current study, histiocytes were primarily increased in those with PE caused by malignant tumors (50.7% of all exudative HisPE). The mechanism of histiocytosis in malignant PE seems to be associated with the immune response to cancer (cancer cells as antigens) [10].

Further, in our study, lung cancer was the most common cause of malignant PE (41.2%), followed by breast cancer (15.7%) and stomach cancer (10.8%). It is reported that lung cancer, breast cancer, and lymphoma account for most cases of malignant PE [2, 17]. However, it is rather interesting that stomach cancer was the third most common cause in our study. This is thought to be due to the higher incidence of stomach cancer in South Korea than in other countries [18]. In addition, it is known that malignant PE can occur in all types of lung cancer, but adenocarcinoma is the most common cause [19, 20]. The present study confirmed that adenocarcinoma is the most common cause (81.0%) of lung cancer-induced malignant PE.

Aside from malignancy, in the present study, HisPE occurred in patients with parapneumonic effusion (4.5%) and tuberculous pleurisy (4.5%). These diseases must be differentiated by using a variety of clinical situations. However, our findings provide some insights. In parapneumonic effusion, neutrophils were the second most common WBCs after histiocytes, whereas in tuberculous pleurisy, the most common cell type was lymphocytes. In tuberculous pleurisy, a prominent rise in ADA was also observed. These findings could aid discrimination of the causative disease of HisPE. Moreover, there were multiple cases of PE due to heart failure, liver cirrhosis, or renal diseases (11.9%). However, since we only applied Light’s criteria, approximately 25% of transudative PE could have been misclassified as exudative PE [2]. Further consideration of the serum-pleural fluid protein gradient as well as Light’s criteria would have significantly reduced the proportion of PE due to heart failure, liver cirrhosis, or renal diseases.

This study has some limitations. First, this was a retrospective study. Second, it was conducted at a single center and the sample was small. It is necessary to confirm whether the HisPE characteristics identified herein can be replicated through prospective studies involving a large number of PE patients. In addition, further studies are needed to determine the mechanism by which histiocytes reactively increase in PE.

## Conclusions

In conclusion, the leading cause of exudative HisPE was malignancy, particularly lung cancer. Physicians should consider the possibility of malignant diseases if histiocytes are predominantly present in pleural fluid analysis.

## Data Availability

The datasets used and/or analyzed during the current study are available from the corresponding author on reasonable request.
